# A systematic review of EPDS cultural suitability with Indigenous mothers: a global perspective

**DOI:** 10.1007/s00737-020-01084-2

**Published:** 2020-11-27

**Authors:** Ai Wen Chan, Corinne Reid, Petra Skeffington, Rhonda Marriott

**Affiliations:** 1 School of Psychology, Exercise Science, Chiropractic & Counselling, Murdoch University, Perth, Australia; 2 Victoria University, Melbourne, Australia; 3 Global Health Academy, The University of Edinburgh, Edinburgh, Scotland; 4 Psychology, Exercise Science, Chiropractic & Counselling, Murdoch University, Perth, Australia; 5 Ngangk Yira Research Centre for Aboriginal Health and Social Equity, Murdoch University, Perth, Australia

**Keywords:** Indigenous, Edinburgh Postnatal Screening Scale (EPDS), Systematic review, Perinatal mental health, Acceptability

## Abstract

The Edinburgh Postnatal Depression Scale (EPDS) is used extensively as the “gold standard” perinatal depression and anxiety screening tool. This study contributes to an emerging discussion about the tool’s shortcomings, specifically around cultural suitability for use with Indigenous women. A systematic search was conducted in ProQuest, PsycINFO, MEDLINE (Web of Science), PubMed, Scopus, Informit, and CINAHL research databases, and grey literature. The quality of the body of evidence was assessed using the NHMRC Level of Evidence framework. Three studies supported the cultural validation of the EPDS with Indigenous groups in Canada (*n* = 2) and the USA (*n* = 1). The remaining eleven Australian studies demonstrated that cultural concerns were suggested by either Indigenous mothers, healthcare professionals (Indigenous and non-Indigenous), or both, though cultural concerns were more weighted from the perspectives of healthcare professionals. The quality of the evidence was not strong, and thus, there is a critical and urgent need for targeted research in this area. This review identified and recommended Indigenous-specific methodologies that can be adopted for more trustworthy, culturally safe, and effective research in this area. Given that the EPDS is currently considered gold standard in routine perinatal mental health screening practice in countries around the world, these findings raise significant concerns. Using culturally relevant research methodologies, such as the use of mixed-methods design, could lay stronger groundwork for further investigation of the broader utility and cultural relevance of the tool.

## Introduction

Symptoms of perinatal depression are sometimes mistaken as typical pregnancy responses (physiological, biological/hormonal, and emotional). Unrecognised and missed diagnosis can result in repercussions like maternal death and stillbirth (Eastwood et al. 2017; Milgrom and Gemmill 2015; National Institute for Health and Care Excellence 2014). Perinatal depression is also associated with health risks and diminished wellbeing of the mother and her family system, such that stress and depression during and after pregnancy can negatively affect an infant’s future growth and development (Cao-Lei et al. 2017; Cook et al. 2018; Gentile 2015, 2017; Goodman 2019).

Globally, Indigenous^1^ women and children typically have worse physical and mental health outcomes compared to non-Indigenous counterparts (Jorm et al. 2012; Lima et al. 2019; Trovato and Romaniuk 2014) and continue to experience inadequate and substandard care and treatment due to marginalisation from mainstream healthcare services (Harfield et al. 2018; McIntyre et al. 2017; Minority Rights Group International 2016; World Health Organization (WHO) 2013). The health gap is further perpetuated by intersecting forces of socio-economic and political experiences resulting from European colonisation including intergenerational trauma, racial discrimination, cultural disconnection and suppression (i.e., loss of language), systematic oppression and assimilation, loss of control (i.e., land loss and home dispossession), and limited access to basic adequate services like health, education, and employment (Anderson et al. 2016; Aguiar and Halseth 2015; Jackson Pulver et al. 2010; Brave Heart et al. 2011).

The most widely used screening tool for identifying women who may be at risk for depression after childbirth is the Edinburgh Postnatal Depression Scale (EPDS; Cox et al. 1987). Originally developed and validated with a sample of postnatal women in Scotland, the use of EPDS has gained widespread acceptance and extensive use over the ensuing 30 years evidenced by its ubiquitous translation and validation (Alvarado et al. 2015; Cox et al. 2014; Department of Health, Government of Western Australia 2006; Hewitt et al. 2010; Joshi et al. 2020; Rhee et al. 2018; Shrestha et al. 2016). However, in recent years, there has been emerging discussion around the limitations of the EPDS, including questioning of the cultural suitability and validity when applied in Indigenous or cross-cultural contexts (Black et al. 2018; Gausia et al. 2013; Geia et al. 2013; State of Queensland 2014). A recent systematic review reported a lack of research investigating the effectiveness of the standard EPDS with Indigenous Australian women, providing neither psychometric properties nor qualitative validation of the standard EPDS (Kotz et al. 2020).

Arguments about cross-cultural validity include questions about the relevance of some items, language barriers, and differences in conceptualisations of mental health (Brave Heart et al. 2011; Douglas 2013; Guerin and Guerin 2012; Kozinszky and Dudas 2015; Laungani 2000). Evidence of cross-cultural inconsistency in cut-off scores have been identified as supporting these claims (Matthey et al. 2006). Variable cut-off scores could be attributed to the different definitions of depression based on the diagnostic criteria and systems used (Gibson et al. 2009). It may also reflect cultural variation in the expression, manifestation, and representation of depressive and anxious symptoms (Oates et al. 2004). Irrespective of origin, stark disparities in validated cut-off scores call into question the level of confidence in EPDS as a screening tool, yet clinical practice guidelines continue to be guided by validated cut-off scores to assist in reviewing and monitoring, and providing supportive referral pathways for women (Department of Health 2019; McCabe-Beane et al. 2016). Variable cut-off scores may indicate cultural differences, reinforcing the need for a strong emphasis on cultural context across validation studies. Developing an evidence-informed understanding of applicability of the EPDS within Indigenous contexts is a high priority to ensure access to services and well-targeted service provision in this high-risk population.

The primary aim of this systematic review is to elucidate how culturally suitable the EPDS is and how variations of EPDS implementation have been used with Indigenous populations across the world. To date, no such reviews exist. Reviewing international literature within this context facilitates shared learning and is undertaken with an intention to empower Indigenous communities when faced with engagement in mainstream health systems that may not be designed to best meet their needs.

## Methods

### Definitions

#### Indigenous

There is no universally accepted definition of “Indigenous” as Indigenous groups, nations, and/or tribes have heterogeneous and diverse definitions and preferred names. For the sake of this review, the definition of “Aboriginal” or “Indigenous” include persons or a group of people who are native to and the original inhabitants of the land or country and possess understanding of these natural environments before the arrival of settlers^2^ (United Nations 2014).

#### Perinatal period

The perinatal period includes the time of gestation when a baby is conceived until after the baby is born, with varying definitions of what constitutes the length of time after baby is born (American Psychiatric Association 2013; Australian Institute of Health and Welfare 2014; WHO 2016). A timeframe was not specified in the search.

### Screening process

On 19 January 2020, a combination of search terms (see Table 1) was used to systematically search the following electronic databases: ProQuest, PsycINFO, MEDLINE (Web of Science), PubMed, Scopus, Informit (including Indigenous Collection), and CINAHL. The search term “Edinburgh Postnatal Depression Scale” was not included because this limited the search results. Additional sources like grey literature were included to maximise the coverage of a wider depth and breadth of existing Indigenous health literature (Derrick et al. 2012). At every opportunity, original authors of studies were directly contacted in an attempt to gain access to additional and relevant information, though this strategy was not always successful.

**Table 1:** Search terms

Indigenous	Aborigin* OR Indigen* OR “Indigenous people*” OR “ethnic minorit*” OR “ethnic group*” OR “minorit* group*” OR native OR “native nation*” OR “First Nation*” OR “First people*” OR “native people*” OR “native population*” OR trib* OR “native trib*” OR “American India*” OR “Alaska* Native*” OR “Native Alaska*” OR “Native America*” OR “Native Hawaii*” OR “Plain India*” OR “Torres Strait” OR Metis OR Inuit OR Eskimo OR Aleut OR Yupik OR Maori OR Sami OR Nenet* OR Komi OR Circassian* OR Polynesian* OR Melanesian* OR Micronesian* OR Papuan*
Perinatal	perinat* OR pregnan* OR antenat* OR prenat* OR postnat* OR prepart* OR postpart* OR peripart* OR antepart* OR intrapart* OR prepart* OR neonat* OR puerper* OR matern*
Depression	depress* OR anxi* OR mood* OR “affective disorder” OR “mental health”

Studies that met the following criteria were included:

1. The study was undertaken after the EPDS was developed and published in 1987 (Cox et al. 1987).
2. EPDS was a primary focus of measurement.
3. The relationship between Indigenous participants and EPDS scores were discussed in detail.
4. Provided evidence and discussion regarding cultural issues and perspectives towards the EPDS.
5. Cross-cultural approaches, strategies, and/or Indigenous research methodologies were used.

Peer-reviewed studies, intervention studies, observational studies, and case reports published in peer-reviewed journals were considered. Study protocols, editorial commentaries, discussion papers, and other variation of an opinion/review papers were excluded.

**Fig. 1:**
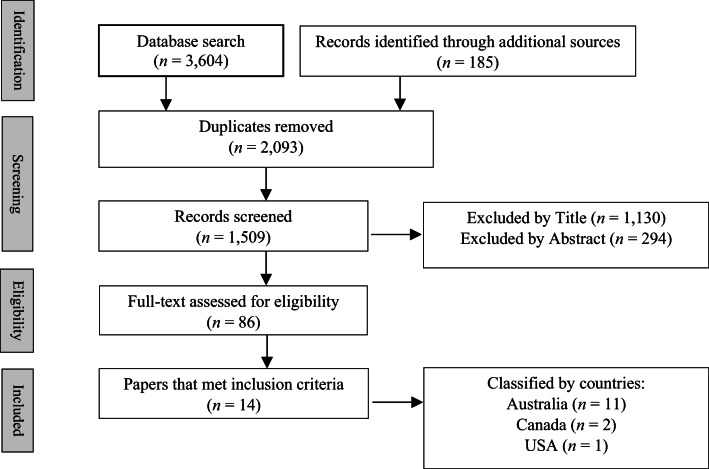
PRISMA flow chart (Moher et al. 2009) showing identified and screened papers to be assessed for eligibility and final inclusion of the review

After duplicates were extracted, 25% of those studies were further independently screened by a co-author (PS) to confirm final eligibility of studies. These studies were selected through a random function on Excel to ensure inter-rater reliability was met by two reviewers (Waddington et al. 2012). Agreement between WC and PS was strong, *r* = 0.939. Eligible studies that were included are shown in Fig. 1.

### Final eligibility of studies

A total of 14 published articles met inclusion criteria. Each stage of the search and screening processes are shown in the Preferred Reporting Items for Systematic Review and Meta-Analysis (PRISMA) flow diagram (Fig. 1).

### Quality appraisal criteria

Studies were ranked according to the National Health and Medical Research Council (NHMRC) levels of evidence (National Health and Medical Research Council, 2009a, b). To accommodate grey literature, a level of evidence based on expert opinion (NHMRC 2000) was added: “Level V: Evidence obtained from expert opinion from clinicians, authorities and/or reports of expert committees”.

## Results

Studies were categorised by country, then ordered by the NHMRC level of evidence ratings (Table 2). Eleven studies were conducted in Australia, two in Canada, and one in the United States of America (USA). All selected studies expressed some degree of cultural concern about the EPDS from either mothers’ and/or healthcare professionals’ (HCP) point of view.

**Table 2:** Characteristics of included studies

Country	Publication	NHMRC level of evidence	Method	Evidence of cultural concerns regarding EPDS
Australia	Freeman et al. 2017 New South Wales	Level III-3 Reported OR > 1, *p* ≤ 0.05, increased risk of postnatal service affecting EPDS acceptance Unreported sample size calculation	Retrospective cohort study (prognosis) 424 Aboriginal children and their mothers (*n* = 215)	Women: yes. 57% (152 of 267 offered) rejected the EPDS. Increased frequency of CFHN interaction positively associated with mother accepting EPDS screening. HCP: unreported.
	Gausia et al. 2013 Nation-wide	Level III-3 Reported OR > 1, *p* < 0.05, Aboriginal, birthing and care plans, financial, < 4 antenatal visits increased risk of missing emotional wellbeing screening	Retrospective cohort study (prognosis) 36 primary health centre (all Australian states) 674/797 pregnant Aboriginal women	Women: unreported. HCP: yes. Language perceived as a concern for Aboriginal mothers. Midwives reported women required assistance when completing the EPDS (anecdotal).
	Hayes et al. 2010 Queensland	Level III-3 EPDS > 9 (not at risk) vs EPDS > 12 (at risk)	Cross-sectional case-control study 92/110 Aboriginal mothers	Women: yes. Application of the translated TAIHS EPDS. HCP: yes. Reference groups revised and altered specific words or simplification of sentence structure.
	WAPMHU and WNHS 2011 Western Australia	Level III-3 Used baseline as comparison point after the service was implemented Over multiple time-points (32 months)	Longitudinal mixed-methods, comparative interrupted time series 34 Aboriginal women 14 HCP	Women: unreported. HCP: no. Midwives perceived that mothers genuinely responded to the EPDS.
	Campbell et al. 2008 Queensland	Level IV Did not use reference standard Reported *z*-scores, 9 women did both measurements at both time-points	Case-series validation study (diagnostic accuracy) 210 Aboriginal women	Women: yes. TAIHS and MIT EPDS modified to be meaningful for women. HCP: yes. Words and sentence structures altered by reference group.
	Highet and Goddard 2014 Nation-wide	Level IV Unreported total *n* of HCP surveyed Cross-sectional, undetermined casual relationship	Mixed-methods, interviews 82 services surveyed, 26% HCP were Aboriginal 28 HCP interviewed, 35% were Aboriginal	Women: unreported. HCP: yes. Some HCP thought EPDS was not meaningful to use, insulting, and “white fella ask questions”.
	Marley et al. 2017 Western Australia	Level IV Used GP diagnosis Cross-sectional, undetermined casual relationship	Case-series validation study (diagnostic accuracy) 97 Aboriginal women 15 HCP	Women: yes. 98% accepted KMMS. unreported % accepted EPDS. HCP: yes. 8/9 HCP reported KMMS was more useful and superior to the EPDS.
	Carlin et al. 2019a Western Australia	Level V Consultations with women with a recent experience of receiving perinatal care in the Pilbara region	Qualitative yarning methodology 15 Aboriginal perinatal women	Women: yes. Supported KMMS more than EPDS. HCP: Unreported.
	Hayes et al. 2005 Queensland	Level V Consultations with reference group members (5 TAIHS, 12 MTI, 5 PI)	Expert opinions 141 antenatal women 127 postnatal women	Women: yes. Piloted translated EPDS versions. HCP: yes. Reference groups modified language, cultural images and colours that were more meaningful to women.
	Kotz et al. 2016 Western Australia	Level V Consultations with midwives and CHNs, Aboriginal women from community as advisory group	Expert opinions through yarning 100 Aboriginal people (8 language groups) 72 HCP	Women: yes. Selected preference in wording and provided additional suggestions. HCP: yes. Advisory group modified wording and formatting.
	Queensland Health 2013 Queensland	Level V Small working party in North QLD Consultations with state-wide partnerships	Expert opinions 43 HCP (11 Aboriginal health workers, 26 registered nurse/midwife)	Women: unreported. HCP: yes. EPDS culturally inappropriate due to language (30%), not asking the right questions (47%), screener lacking cultural expertise (42%).
Canada	Clarke 2008b Saskatchewan	Level IV Used Clinical Interviews DSM-IV diagnosis Reported OR > 1 but *p* > 0.05	Case-series validation study (diagnostic accuracy) 103 First Nations and Metis women	Women: no. Validated standard EPDS with PDSS and BDI-II. HCP: unreported.
	Clarke 2008a Saskatchewan	Level V Expert opinion, unpublished doctoral thesis Used Structured Clinical Interviews DSM-IV diagnosis	Qualitative interview methods 9 Aboriginal postpartum women	Women: no. Congruence between high EPDS score, diagnosis, and feelings of disconnect with baby. HCP: unreported.
USA	Heck 2018 Nation-wide	Level IV Inclusion criteria was unclear Studies in this review were mainly Level C^1^ Used DSM, SCID, MINI, ICD-10, CIDI, some unreported reference standard	Systematic review of EPDS (*n* = 54) and PHQ-9 (*n* = 7) validation studies with American Indian and Alaska Native (AI/AN) women	Women: no. Validated EPDS with AI/AN women. HCP: unreported.

*OR* odds ratio that quantities the strengths of the relationship between two variables, *CFHN* Child and Family Health Nurse, *TAIHS* Townsville Aboriginal and Islanders Health Services (an Aboriginal community controlled health service in Queensland), *WAPMHU* Western Australian Perinatal Mental Health Unit, *WNHS* Women and Newborn Health Service, *MTI* Mount Isa (city in Queensland), *KMMS* Kimberley Mums Mood Scale, *PI* Palm Island, *CHN* Child Health Nurse, *PDSS* Postpartum Depression Screening Scale, *BDI-II* Beck Depression Inventory-II, *DSM* Diagnostic and Statistical Manual of Mental Disorders, *SCID* Structured Clinical Interview for DSM, *MINI* Mini International Neuropsychiatric Interview, *ICD-10* The International Classification of Diseases, 10th Revision, *CIDI* Composite International Diagnostics Interview, *PHQ-9* Patient Health Questionnaire-9

Level C of qualitative studies, descriptive, correlational, integrative reviews, and systematic review/RCT with inconsistent results on AACN Levels of Evidence (Armola et al. 2009). Heck’s (2018) review consisted of Level 3 studies on American Association of Critical-Care Nurse’s Evidence-Leveling System (Armola et al. 2009), equivalent to a low rating of NHRMC III-3 and IV level of evidence

### Assessing quality of body of evidence

Due to the heterogeneity of research methodologies, interventions, and outcomes in the eligible studies, a quantitative summary measure of the results was not applied. Instead, a descriptive analysis of the selected publications enabled more robust comparisons between studies’ findings. The synthesis of evidence was weighed transparently, critically addressed, and reviewed according to the National Health and Medical Research Council (NHMRC) framework (NHMRC 2009b). The following ratings assigned for each component will inform future research and recommendations.

#### Evidence base

The quality of the body of evidence was not high—it was constrained by the lowest Level V studies, with Level III-3 (*n* = 5/14) being the highest. A Poor (level D) quality of evidence was determined.

#### Consistency

The majority of studies (*n* = 10) conveyed cultural concerns from Indigenous women and/or healthcare professional (HCP) in relation to the standard EPDS, resulting in a Satisfactory (level C) rating.

#### Clinical impact

Variable results suggested that the standard EPDS was not suitable for Indigenous Australian mothers; hence, a rating of Good (level B) was given as the studies presented strong evidence of clinical relevance (challenging current practice) and potentially positive clinical impact if these findings are taken into account when reviewing the use of measures of postnatal depression for Indigenous contexts.

#### Generalisability

Selected studies were predominantly conducted in Australia (*n* = 10), and of that, only two studies sampled participants nation-wide. Several studies were specific to the regions in which the studies were conducted, like Townsville (QLD), Kimberley (WA), Pilbara (WA), and Saskatchewan (Canada). Hence, a Poor (level D) was applied.

#### Applicability

The evaluated evidence was rated Satisfactory (level C). The existing differences in findings, perspectives, and recommendations highlighted in this review should not hinder direct application of studies’ findings when considered with Indigenous Australian populations.

The overall body of evidence was at a poor to satisfactory level, which constrains any conclusions that can be drawn about EPDS use and highlights the urgent need for further research in this area. There are several key findings methodological from the current study that can helpfully inform planning for future research.

### Key findings

#### Psychometric validation

In a prospective study, it was felt that congruence between a woman’s DSM-IV diagnosis and her high EPDS score was validated by her feelings of disinterest in her baby during pregnancy, not bonding with her child at birth, and feeling overwhelmed during postpartum (Clarke 2008b). Two other studies found that, according to this psychometric validation, the standard EPDS was suitable for use with First Nations and Metis women (Clarke 2008b) and American Indian and Alaska Native (AI/AN) women (Heck 2018). However, instruments like the PDSS and BDI-II were used in these studies to validate the EPDS (Clarke 2008b), yet these have not themselves been validated with Indigenous populations, and cultural differences in preferences, biasses, and interpretations of mental health were noted (Heck 2018). This “chicken and egg” quandary needs to be resolved in Indigenous validation studies—a mixed methodology involving “blind” review by local Indigenous health workers is one potential solution, and inviting mothers and healthcare workers to reflect on their experience of using the EPDS is another.

#### Perceptions and expressed concerns

Three qualitative Australian studies reported views from the HCP’s perspective, indicating mixed results. Some found that HCP had low regard for EPDS scores when used with Indigenous women (WAPMHU and WNHS 2011), others found the EPDS was not a meaningful measure due to language barriers (Gausia et al. 2013) and that it was insulting and culturally inappropriate, whilst others felt that it was an acceptable measure (Highet and Goddard 2014). A Queensland-wide survey reported 37% of clinicians raised concerns about the EPDS not accurately identifying issues and 58% clinicians reported that the EPDS lacked cultural relevance (Queensland Health 2013).

### Actions speak louder than words: looking for incidental indicators

#### EPDS rejection

Taking a population health approach to the issue of cultural validity, one Australian study found that the reported overall rate of women completing the EPDS was lower than those who rejected EPDS screening (43% vs 57%, *n* = 115) and drastically lower than the rate of completion by mothers in the general population 98% (Freeman et al. 2017). The lack of discussion of underlying reasons for EPDS rejection was a significant design limitation, though a higher EPDS rejection rate is telling in itself. These findings also highlight the potential for biassed sampling in validation studies. Since only a minority of women are turning up for screening, it can be argued that those who do not attend may be a more high-risk sample and critical for validation of the instrument.

#### Cultural translations and adaptations

Cultural concerns around standard EPDS suitability are implicit in the decision to translate the EPDS into the Townsville Aboriginal and Islanders Health Services (TAIHS) version (Campbell et al. 2008; Hayes et al. 2005; Hayes et al. 2010) and adapt the EPDS to form the Kimberley Mums Mood Scale (KMMS) (Carlin et al. 2019a; Kotz et al. 2016; Marley et al. 2017). Perinatal Aboriginal women in the Pilbara region in WA supported the KMMS and preferred it over the EPDS because of its simplified language and yarning element of KMMS (Carlin et al. 2019a).

These modifications and translations address perceived wording and formatting limitations (Kotz et al. 2016) and reflect more implicit and common usage of words in the local dialect (Hayes et al. 2010) as well as the use of face emoticon in place of a numeric Likert scale. Studies using translated or adapted EPDS versions demonstrated an intended movement towards a more culturally safe screening process for Indigenous women and families (Hayes et al. 2010; Marley et al. 2017); however, there have also been some challenges to the validity of these instruments, for example, Hayes et al. (2005) found a response bias in which women persistently selected the happiest emoticon face.

#### Modified approaches to implementing EPDS

A mixed-methods questionnaire revealed that Australian HCPs working with Indigenous mothers described adaptations to the implementation of the EPDS—specifically, using the EPDS in an informal, conversational way (Highet and Goddard 2014). Some HCPs (21%) weave the EPDS questions into conversation, whilst an alternative approach to EPDS implementation was to provide assistance (Gausia et al. 2013), modify the words to suit the client and to add prompts about family involvement (i.e., partner), and support networks (Highet and Goddard 2014). Part II of the KMMS was designed to address these issues by opening a discussion around additional emotional, social, or physical health concerns or risk factors (Marley et al. 2017). As well as implementing Part II of KMMS, the kinds of questioning and how the HCP approached such sensitive questions were critically important too (Carlin et al. 2019a). Australia’s perinatal best practice guidelines currently state that language and cultural appropriateness of the EPDS should be considered when screening with Indigenous women, such as adapting the way the questions are asked (Austin et al. 2017), though it is not clear how this should be achieved.

#### Culturally relevant research methodology

Studies describing the process of tool development (TAIHS, KMMS) outlined the importance of being patient, providing time and space to listen to and seek endorsement from Indigenous women with respect to preferences or suggestions for wording, emerging layout themes, and implementation management and strategies (Campbell et al. 2008; Hayes et al. 2005; Kotz et al. 2016; Marley et al. 2017). Allowing Indigenous women and communities to have a voice is necessary in building strong partnerships with Indigenous communities (Campbell et al. 2008; Carlin et al. 2019a; WAPMHU and WNHS 2011). Possible strategies to enhance community engagement and participation is the use of yarning technique (Carlin et al. 2019a; Kotz et al. 2017) or the use of Indigenous interviewers (Clarke 2008a). Yarning, as a research methodology, follows an open-ended and conversational approach that acknowledges Indigenous principles of oral language, story-telling, and shared experiences (Bernardes et al. 2020; Walker et al. 2014). The method of yarning is essential in the research process as it highlights reciprocity between the participant and researcher and is based around principles of respect, empowerment, and cultural sensitivity (Smith et al. 2020). Qualitative methods, similar yet distinguishable to the yarning style, uncovered rich experiences of using the EPDS that was complementary to the EPDS score itself (Carlin et al. 2019a; Clarke, 2008a; Highet and Goddard 2014; Marley et al. 2017; WAPMHU and WNHS 2011).

## Discussion

The cultural acceptability of the EPDS within an Indigenous context remains unclear and unresolved. Resolution of this issue is a high priority given the disturbingly poor health and wellbeing outcomes for Indigenous mothers and babies. Together, these studies have identified methodological limitations and, thus, suggest a potentially stronger focus on methodological design and approaches when addressing cultural security issues in future research.

### Questioning current EPDS implementation

Findings continuously indicate that Indigenous women are being less likely to be screened than non-Indigenous women (Gausia et al. 2013; San Martin Porter et al. 2019), perhaps because Indigenous women may be less likely to attend antenatal appointments (San Martin Porter et al. 2019) or that Indigenous women may be less likely to accept the invitation to complete the EPDS (Freeman et al. 2017). Studies identified barriers to EPDS screening such as issues around fear and stigma (Highet and Goddard 2014), whilst other studies highlighted enablers such as trust, engagement, and ongoing commitment with Indigenous groups (Freeman et al. 2017; Kotz et al. 2016; WAPMHU and WNHS 2011). However, even with the newly developed translations and adaptations of the EPDS, findings of suitability (i.e., cut-off points, validity) were inconclusive. Adaptive approaches to EPDS implementation, like deeper inquiry and further discussions rather than the traditionally structured question-and-answer approach to screening, may have positive implications for engagement but also potentially have negative implications for validation and moreover, for the way the EPDS results are interpreted clinically and in research studies.

Notably, whilst gaining international use in clinical and research work over the years, the EPDS has been used in potentially substandard and misleading ways that departed from the original purpose. The original authors and developers clarified that the EPDS should not be used as a diagnosis assessment, but rather as part of screening process that facilitates “discussing her responses and listening to her story” (Cox 2017, 2019). Considering the low-quality evidence of this current systematic review, researchers and clinicians should understand, refer back, and adhere to the original intent of the EPDS.

Finally, due acknowledgement is required to the unique differences in interpretation and perceptions of emotional mental health and manifestation of postpartum depression and anxiety across Indigenous groups in America (Heck, 2018) and Indigenous Australian populations (Buist et al. 2007; State of Queensland 2014). For instance, “social and emotional wellbeing” (SEWB) is preferred by Indigenous Australians because of its holistic, emotional, and social connotations of health (Gausia et al. 2015). The EPDS fails to consider these differences and can contribute to women answering differently, or to significant aspects of mental wellness not being captured by the tool at all. In light of cultural variations of psychological experiences and cultural norms of mental health conceptualisation, EPDS findings should be interpreted with caution and cultural sensitivity.

Given that neither EPDS nor SEWB measures have been validated with Indigenous Australian groups, it may be that SEWB enquiry can fruitfully supplement EPDS screening for Indigenous Australians women—minimally SEWB inquiry covers well-established risk factors for mental health disorders in Indigenous communities (Gausia et al. 2015). In the absence of a contextually validated measure, more supplementary attention should be paid to the historical, cultural, social, and economic contexts and aspects of mental health.

### Critical reflection: acceptability and validation

Cultural translations, adaptations, and modifications to the standard EPDS have been proposed, developed, and implemented. These may be considered more acceptable perinatal mental health screening practices with Indigenous populations. Currently, acceptability is being prioritised over validation but given that the purpose of perinatal assessment is to use results to guide treatment decisions and resource allocation, both elements of validity should be included together in future studies. Perhaps emphasis could be given to the need for both a culturally acceptable and methodologically robust version of the EPDS to ensure that there is no significant trade-off between these two elements.

Possibly of more importance is that the available studies highlighted the difficulty in developing and evaluating a new measure. There was notably limited evidence of sound psychometric properties of the adapted instruments and little evidence that managed to ascertain whether the EPDS, when used in its standard format, was sensitive to identifying those at risk for perinatal depression in this context. Minimally, improved methodology for adaptations and evaluations warrants exploration and critical reflection.

### Mixed-methods research methodology

Employing culturally relevant research methodologies is a way to honour, respect, and prioritise culture by expressing a genuine partnership with those who are trusted in both cultures (Buist et al. 2007). Gathering stories by yarning is aligned with Indigenous’ oral narrative culture and offers great potential for better understanding women’s experiences (Lin et al. 2016). However, the qualitative studies presented in this review were not methodologically strong. For instance, the qualitative approach in validating the KMMS shed light on its acceptability compared to the standard EPDS (Carlin et al. 2019a; Marley et al. 2017) but it remains unclear whether this measure provides a safe way of understanding the risk of depression in Indigenous mothers. Encouragingly, a more detailed study protocol has been proposed to evaluate and further confirm validation/revalidation and acceptability of the KMMS in the Kimberley, Pilbara, and far north Queensland regions in Australia (Carlin et al. 2019b).

Reciprocal cultural consultation and shared cultural wisdom between reference groups and conversations with community-based Indigenous women was key in facilitating trusted relationships and partnerships with the researchers (Campbell et al. 2008; Carlin et al. 2019a; Hayes et al. 2005; Kotz et al. 2016; Marley et al. 2017). There was general agreement that without trusting collaboration and allies, Indigenous participants and communities will continue to view research as culturally unsafe and as another form of loss of control and ownership (Owais et al. 2020). If trust is hard to gain, and could be even harder to gain when quantitative methods are applied, perhaps qualitative methods could be the preferred path. In addition to the inherent strengths of qualitative methods in this context, the resultant relationships may also lay a stronger foundational groundwork for further quantitative (psychometric) process to be introduced at a later time.

Rather than minimising either the quantitative or qualitative methods, a mixed-methods approach integrating both methodological design may ultimately provide a stronger design (Johnson and Onwuegbuzie 2004). The undertaking of a mixed-methods approach that focuses on building rapport and trust with Indigenous women may be more culturally effective in encouraging Indigenous research participation and community engagement. Furthermore, process-oriented documentation and reflective reporting in research should be considered a key form of accountability in order to learn from both successful methodologies, challenges and barriers, and mistakes.

### Limitations of included studies

One significant reason for the poor quality of results may be the variability in implementation of the measures as HCP lacked awareness and knowledge in understanding and interpreting the EPDS (i.e., using it as a diagnostic tool; WAPMHU and WNHS 2011). Many papers suggested that HCP did not adhere to standard protocol when implementing the EPDS, and also possibly, when implementing other measures. An error in EPDS administration or lack of accuracy introduces nonstandardised error variance to case identification using the EPDS tool, questioning the clinical impacts this has on clients as well as undermining interpretation of research findings and collecting accurate health data (Di Florio et al. 2017).

Notably, cultural concerns about the EPDS were derived primarily from perspectives of HCP and not confirmed by women, so inferences about cultural concerns were tentative and not strong in quality. For example, underlying reasons for EPDS rejection were not further explored or discussed in detail (Freeman et al. 2017). Whilst there is reported evidence of Indigenous mothers who have accepted either the EPDS or an alternative measurement/assessment, it remains unclear whether participants avoided screening because of the EPDS as a tool or because of a wider issue regarding stigma of mental health screening in general.

Those that included both perspectives were studies that translated and adapted the standard EPDS. Yet, these studies presented methodological limitations including poor psychometric validation and lack of generalisability. Together, these limitations pose a risk for accuracy of case identification using the EPDS and significantly restricts the confidence in the interpretation of the findings.

Clinical anxiety indicators of the EPDS were not extensively discussed within the international Indigenous literature, and where it was discussed, anxiety disorders were discussed in conjunction with depressive disorders rather than as a standalone feature. The EPDS anxiety subscale (items 3, 4, and 5) has been suggested to screen for anxiety in perinatal women (Bowen et al. 2008; Grigoriadis et al. 2011; Matthey 2008; Matthey et al. 2013). Indigenous women commonly experience anxiety as mothers-to-be and have additional stresses of other complex and multifaceted factors and vulnerabilities that come into play in relation to mental health and wellbeing (Carlin et al. 2019a; Parker and Milroy 2014). To avoid missed opportunities for case identification, future studies could determine a distinction between anxiety and depression when enquiring about psychosocial factors by means of considering the EPDS anxiety subscale as a separate score from the total EPDS score (Jomeen and Martin 2005).

In sum, validation studies remain critically needed in an international context. The absence of culturally valid referent measures remains a challenge—a step-by-step process is required.

### Limitations of this review

This review attempted to include a wider global perspective of the cultural suitability of the EPDS used with Indigenous populations. We found a concerningly limited evidence-base. The majority of included studies focused on Indigenous communities in Australia, with only three of 14 studies from the USA and Canada. There were no available studies that included Indigenous populations in other geographical regions like New Zealand, Europe, Africa, South America, Pacific Islanders, and the Polynesian and Artic countries. This lack of global evidence-base for use of the EPDS with Indigenous communities is a particularly important finding given the ubiquity of use of the EPDS around the world.

The scope of this review focused on the cultural suitability of EPDS with Indigenous women. Studies that simply reported prevalence of perinatal depression or depressive symptoms (Daoud et al. 2019; Nelson et al. 2018; Owais et al. 2020; Stock et al. 2013; Theme Filha et al. 2016) and thoughts of self-harm during pregnancy (Shah et al. 2011) as indicated by the EPDS were not included as cultural views of the EPDS were not discussed. Unexpectedly but equally important, the focus of culturally sensitive research methodology was an essential component of this review. For example, a study that triaged mothers who entered the emergency department at a paediatric hospital was not considered a culturally robust and comprehensive methodology to recruit an Indigenous sample where Indigenous women were poorly represented and were recruited by chance (Stock et al. 2013). The EPDS has also been researched with other Indigenous groups like Māori people in New Zealand (Waldie et al. 2015). Studies as such were excluded from the review but could potentially provide evidence of EPDS usability in other settings or contexts that are not within the scope of this review.

As described, studies were also heterogeneous in their design and methodologies were of variable quality making it hard to combine, compare, and synthesise findings. The disunity in findings between countries and within countries may reflect actual differences or may be a methodological artefact. There were studies that reported either from HCP’s or women’s points of views. Although understanding HCP’s views shed light on the research question, hearing from a third person’s view of whether the EPDS is deemed suitable for women does not constitute privileged evidence. Triangulation of multiple lines of evidence fosters a strongest inference through a process of looking at points of congruence in evidence (Creswell and Plano Clarke 2018; Thurston et al. 2014).

Finally, whilst there were two raters working both independently and collaboratively on identifying, extracting, and synthesising findings, one rater predominantly took lead on the narrative synthesis of findings, whilst the second rater supported and discussed the work as it progressed. It was unknown whether using a different research team or more reviewers would yield different results (Lucas et al. 2007).

## Conclusion

A review of the literature was carried out to synthesise available evidence on whether the use of a widely accepted perinatal screening instrument (EPDS) is culturally suitable for Indigenous people across the world. Each study had different aims, methodology, and conclusions, and the quality of methodology was poor when considered against the NHMRC criteria, and therefore, results were inconclusive. However, findings highlight some significant concerns and underline a clear clinical urgency for more robust and culturally secure and stable research to ensure that our screening measures are helpful for this at-risk group of mothers.

## Footnotes

1 This paper uses the term “Indigenous” to describe first inhabitants or first claims of land, territory, or country. This term has gained acceptance to use within an international context as it includes peoples who have experienced similar colonising circumstances (Peters and Mika, 2017).

2 This definition is not intended to destroy the heterogeneity and diversity of Indigenous groups and tribes and is not an attempt to overlook preferred names by specific nation or tribes.
